# Point-of-Care Ultrasound in Detection, Severity and Mechanism of Significant Valvular Heart Disease and Clinical Management

**DOI:** 10.3390/jcm12206474

**Published:** 2023-10-11

**Authors:** Songnan Wen, Tasneem Z. Naqvi

**Affiliations:** Department of Cardiovascular Medicine, Division of Echocardiography, Mayo Clinic, 13400 East Shea Boulevard, Scottsdale, AZ 85259, USA; wen.songnan@mayo.edu

**Keywords:** point-of-care ultrasound, echocardiography, valvular heart disease

## Abstract

Background: Early diagnosis of significant valvular heart disease (VHD) enables appropriate implementation of the best therapeutic strategy and follow-up. Cardiac auscultation remains suboptimal in early detection of VHD. The aim of this study was to evaluate the utility of point-of-care ultrasound (POCUS) for early detection of VHD and its severity. Methods: All consecutive patients with VHD who did not have a standard echocardiogram prior to first outpatient cardiology consultation underwent history and physical examination followed by POCUS study by an experienced physician in a general cardiology clinic from June 2017 to August 2022 at our institution. Subsequent standard transthoracic echocardiography (sTTE) was performed as the gold standard. Comparison was performed between POCUS and sTTE for the presence and severity of VHD. sTTE was performed by registered cardiac sonographers and interpreted by another cardiologist blinded to the POCUS results. Results: A total of 77 patients were studied (ge 72 ± 11 years, 58.4% males). A total of 89 significant valvular abnormalities were diagnosed. There were 39 (43.8%) cases of regurgitant VHD, 16 (18.0%) of stenotic VHD and 34 (38.2%) had evaluation for prosthetic valve function. The sensitivity (90.9%; 82.4%; 83.3%; 100%) and specificity (100%; 96.7%; 100%; 100%) were high for detecting ≥ moderate aortic regurgitation (AR), mitral regurgitation (MR), aortic stenosis (AS) and prosthetic valvular abnormality, respectively. The weighted κ coefficient between POCUS and sTTE for the assessment of ≥ moderate MR, AR and AS was 0.81 (95% CI, 0.65–0.97), 0.94 (95% CI, 0.84–1.00) and 0.88 (95% CI, 0.76–1.0), respectively, indicating excellent agreement. Conclusions: POCUS can identify patients with significant VHD and may serve as a powerful screening tool for early detection of significant VHD in the outpatient clinical practice with downstream impact on clinical management of significant VHD.

## 1. Introduction

Valvular heart disease (VHD) is a common problem in clinical practice and is a leading cause of mortality and morbidity. The burden of mortality and morbidity associated with VHD is expected to increase worldwide due to increasing life expectancy [1,2,3,4,5,6]. Unfortunately, many patients with VHD do not receive early diagnosis or optimal guideline-based clinical care [4,7]. Waiting until more symptoms appear can delay the opportunity for early intervention to prevent irreversible cardiac remodeling and associated complications. Therefore, early diagnosis of significant VHD is crucial for appropriate follow-up and implementation of the best therapeutic strategy. Currently, standard transthoracic echocardiography (sTTE) is considered the gold standard for diagnosing VHD and assessing its severity and prognosis.

Point-of-care ultrasound (POCUS) has experienced significant growth in recent decades. This is due to advancements in technology, which have allowed for rapid diagnostic evaluation and the performance of various bedside procedures [8]. Previous studies have demonstrated that POCUS has a high sensitivity and specificity in detecting moderate VHD [8]. A recent study [9] further supports the reliability of POCUS in identifying significant left VHD using a pocket-sized device, suggesting it as a potential new screening tool. However, the authors did not attempt to classify the degree of VHD (mild, moderate, severe) due to the absence of spectral Doppler. In our study, we aimed to assess the usefulness of POCUS with a small ultrasound platform during cardiac consultations conducted by an echocardiologist. We compared the findings of POCUS with those of sTTE in detecting the presence and severity of VHD.

## 2. Methods

### 2.1. Study Population

The study was approved by the Mayo Clinic Institutional Review Board, and all patients gave consent for use of their data for research purposes. The study population comprised consecutive patients who were seen for their first cardiology consultation at our center by a single level III echocardiologist (TZN) between June 2017 and August 2022, who had not undergone a prior echocardiogram, and in whom the echocardiologist performed a POCUS study for suspected VHD after history and physical examination (and review of outside medical records if available) during the cardiology consultation.

### 2.2. POCUS

All POCUS imaging was performed using a Philips CX50 portable ultrasound machine, which was equipped with two-dimensional (2D) B-mode, M-mode, color Doppler, pulsed-wave (PW) and continuous-wave (CW) Doppler, and an S5 1-MHz-phase-array pure wave crystal transducer with a resolution of 1400 × 1050 pixels. Images were obtained in the left lateral decubitus and supine positions from the patient’s left side using an echo bed with a drop-down. The images were stored in Digital Imaging and Communications in Medicine format on the local hard disk of the device and transferred to PACS via internet. The ultrasound examination protocol was flexible and goal-directed, and included parasternal long axis (PLAX); right ventricular inflow long axis; parasternal short axis at the aortic valve, mitral valve and papillary muscle level; apical 4–5–3-chamber; subcostal long axis and inferior vena cava views, and suprasternal views for aortic stenosis (AS). These POCUS images were interpreted live by the echocardiologist during patient consultation and dictated with consultation notes. The change in patient care was based on live interpretation of POCUS images at the time of cardiology consultation. All patients subsequently underwent sTTE for confirmation and availability of images and reporting in the echocardiography platform.

The summary of clinical and echocardiographic findings was retrieved from the cardiologist’s dictated notes. POCUS studies were retrieved and reviewed, and additional measurements were made as needed (SW). The exclusion criterion was inadequate image quality. In patients with moderate or greater degree of VHD, quantitative volumetric estimations were performed, and severity of valvular stenosis and regurgitation was assessed. Qualitative assessment of chamber size, left and right ventricular dysfunction and pericardial effusion (PE) was performed at the time of the examination. The presence of valvular abnormalities (regurgitant or stenotic) and their severity (mild, moderate, or severe) were also evaluated during consultation as per American Society of Echocardiography (ASE) recommended definitions [10].

### 2.3. Standard Transthoracic Echocardiography

All standard complete transthoracic echocardiographic imaging was performed using standard ultrasound platforms (Vivid E9 (2017–2019 and E95 (2019 to 2022; GE Vingmed Ultrasound, Horten, Norway), with images ranging between 130–180 with mean delay of 33 days (75% of patients). sTTE was performed by registered cardiac sonographers, who carried out all standard 2D and Doppler measurements according to ASE guidelines. Stored sTTE images were transferred to a Prosolv (Fuji) image archival system. These images were retrieved, reviewed and interpreted blindly by independent cardiology consultants with level III echocardiography training. Measurements were performed using an Enterprise Image Management System according to standard recommendations [11,12]. Left ventricular (LV) end-diastolic (LVEDD) and end-systolic diameters (LVESD), LV ejection fraction (LVEF, by biplane Simpson’s method) and systolic tricuspid regurgitation (TR) velocity were retrieved from medical reports. Right ventricular systolic pressure (RVSP) was estimated in a standard fashion per ASE guidelines. Valve regurgitation severity was assessed qualitatively as well as quantitatively using proximal isovelocity surface area (PISA) as per ASE guidelines [10]. Valve stenosis severity was assessed by continuity equation.

For both POCUS and sTTE studies, the severity of regurgitant VHD was assessed according to the visual interpretation of the 2D cardiac valve appearance along with color Doppler jet turbulence and size, spectral Doppler of the regurgitant jet, as well as cardiac chamber morphology (atrial and ventricular dimensions) and systolic function. The severity of stenotic VHD was based on the evaluation of valve thickness, calcification and mobility on grey scale, color Doppler aliasing, PW and CW Doppler and cardiac chamber (atrial and ventricular) size and hypertrophy. Significant VHD was defined as ≥ moderate VHD including aortic regurgitation (AR), AS, mitral regurgitation (MR), mitral stenosis (MS), and TR. Prosthetic valve function, whether normal or abnormal, was assessed in patients who had a history of valve repair and/or valve replacement.

### 2.4. Clinical Data

Baseline demographics, comorbidities, medications, and laboratory results (hemoglobin, serum creatinine, and N-terminal pro B-type natriuretic peptide (NT-proBNP), etc.) at the time of index cardiology consultation were retrieved from the electronic medical records.

### 2.5. Follow-Up

Data on clinical management, incorporating POCUS assessment at the time of index consultation, were collected as well as of the follow-up sTTE. Follow-up status was determined from the electronic medical records, autopsy reports, and Social Security Death Index.

### 2.6. Statistical Analysis

Data are expressed as mean ± standard deviation (SD), median (interquartile range, IQR) or as numbers (percentages). sTTE was considered the gold standard for the detection and quantification of VHD. Sensitivity and specificity were calculated to evaluate the accuracy of POCUS in the detection of significant VHD. Cohen’s κ coefficient was used to evaluate agreement on the evaluation of significant VHD between POCUS and sTTE. All analyses were performed using BlueSky Statistics software v. 7.10 (Blue-Sky Statistics LLC, Chicago, IL, USA).

## 3. Results

### 3.1. Study Population

Clinical characteristics of the study population are shown in Table 1, and echocardiographic features are presented in Table 2. The mean age of the study population was 72 ± 11 years, and 45 patients (58.4%) were men. The mean LVEF was 58 ± 11%, and 79.2% of patients were in sinus rhythm during sTTE. A mean of 44 ± 22 POCUS images were collected per patient, and the mean duration of POCUS imaging based on the timer on the ultrasound machine was 11 ± 9 min. A total of 89 significant valvular abnormalities were diagnosed in 77 patients. Regurgitant lesions were predominant, present in 39 cases (43.8%), while stenotic lesions were present in 16 (18.0%) cases. Prosthetic valve function was assessed in 34 (38.2%) cases. In 35 (45.5%) patients, medical treatment was initiated or adjusted during POCUS-assisted consultation. Surgical or percutaneous intervention referral was performed in 17 (22.1%) (Table 3). During a mean follow-up of 2.74 ± 1.52 years, 11 (11/77, 14.2%) patients died. Of them, two died of heart failure, four died after cardiac surgery, one died of cancer and one of subdural hematoma. Cause of death was unknown in three patients. 

### 3.2. ≥Moderate Valvular Regurgitation

**Figure 1:**
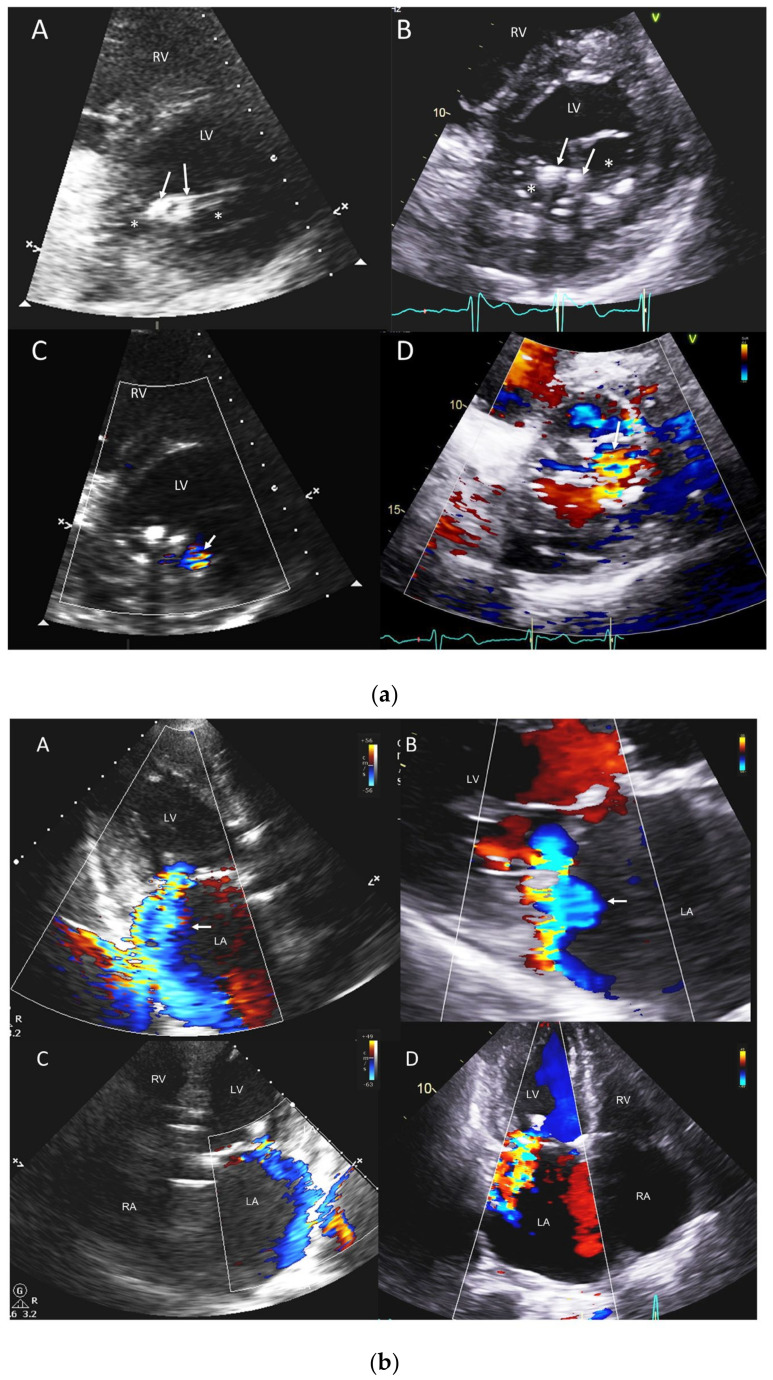
(**a**) POCUS in percutaneous transcatheter-based mitral valve repair. A 79-year-old male with residual MR post transcatheter edge-to-edge repair of the mitral valve (TEER) using mitral clips (2) implanted at an outside facility. POCUS images (left-side panels) compared to the sTTE images (right-side panels). (**A**,**B**) Parasternal short-axis views showing two mitral valve clips (white arrows (**A**,**B**)), a double-orifice mitral valve (white asterisks (**A**,**B**)); (**C**,**D**) color Doppler views at the same level showing origin of MR jet from the anterolateral orifice (white arrows (**C**,**D**)). (**b**) POCUS in percutaneous transcatheter-based mitral valve repair. POCUS sagittal color Doppler images (left-side panels) compared to the sTTE images (right-side panels) in the same patient as in Figure 1, showing significant residual MR post TEER. (**A**,**B**) Apical three-chamber and parasternal long-axis color Doppler views showing severe eccentric MR (white arrows (**A**,**B**)) hugging along the posterior wall (Coanda effect with the MR jet splaying circumferentially along part of the LA wall and shown in a single plane on 2D imaging, thereby underestimating the jet size) of a dilated LA. (**C**,**D**) Apical four-chamber views with LV on the right side on POCUS image (**C**) and on the left side on sTTE image (**D**), with moderate to severe appearing eccentric MR hugging the lateral wall (Coanda effect) of a severely enlarged LA. LA, left atrium; LV, left ventricle; RA, right atrium; RV, right ventricle.

**Figure 2:**
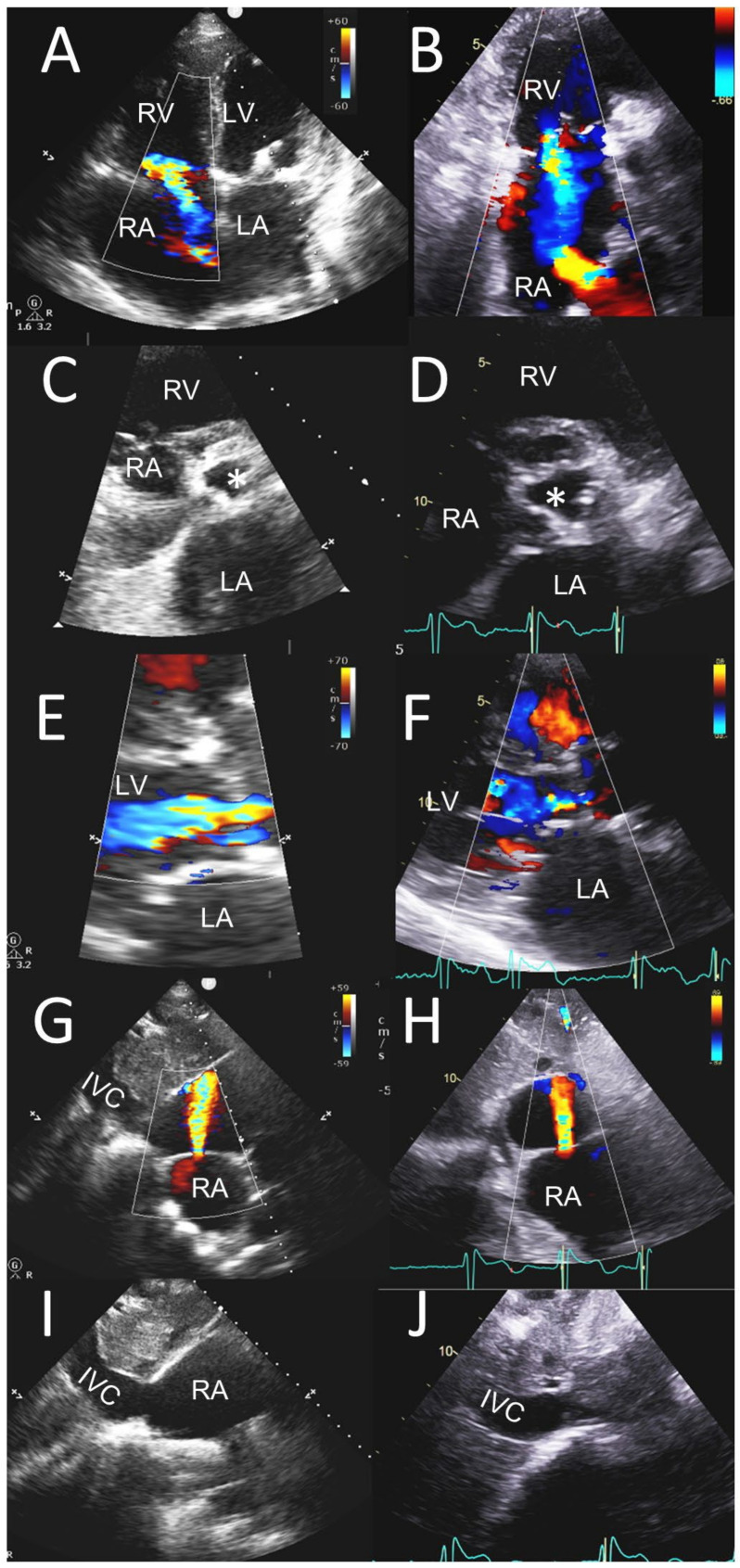
POCUS in assessment of chamber size, shunts and native VHD. Significant TR, AR and other findings. POCUS images (left-side panels) compared to the sTTE images (right-side panels) in the same patient as in Figure 1. (**A**,**B**) Tricuspid inflow views in four-chamber view (**A**) and basal short-axis view (**B**) showing moderately dilated RV and severely enlarged RA (**A**) with moderate appearing TR jet (**A**,**B**) and left-to-right flow across mid interatrial septum from prior trans-septal puncture during TEER (**B**). (**C**,**D**) Parasternal short-axis views showing a moderately sclerotic trileaflet aortic valve in systole (white asterisks) with enlarged LA. (**E**,**F**) Parasternal long-axis views showing AR jet appearing mild to moderate in panel (**E**) and mild in panel (**F**). (**G**,**H**) Subcostal views showing dilated LA and RA and interatrial septum with left-to-right interatrial shunt though the center of interatrial septum from prior TEER. (**I**,**J**) Subcostal sagittal views showing IVC. IVC, inferior vena cava; LA, left atrium; LV, left ventricle; RA, right atrium; RV, right ventricle.

**Figure 3:**
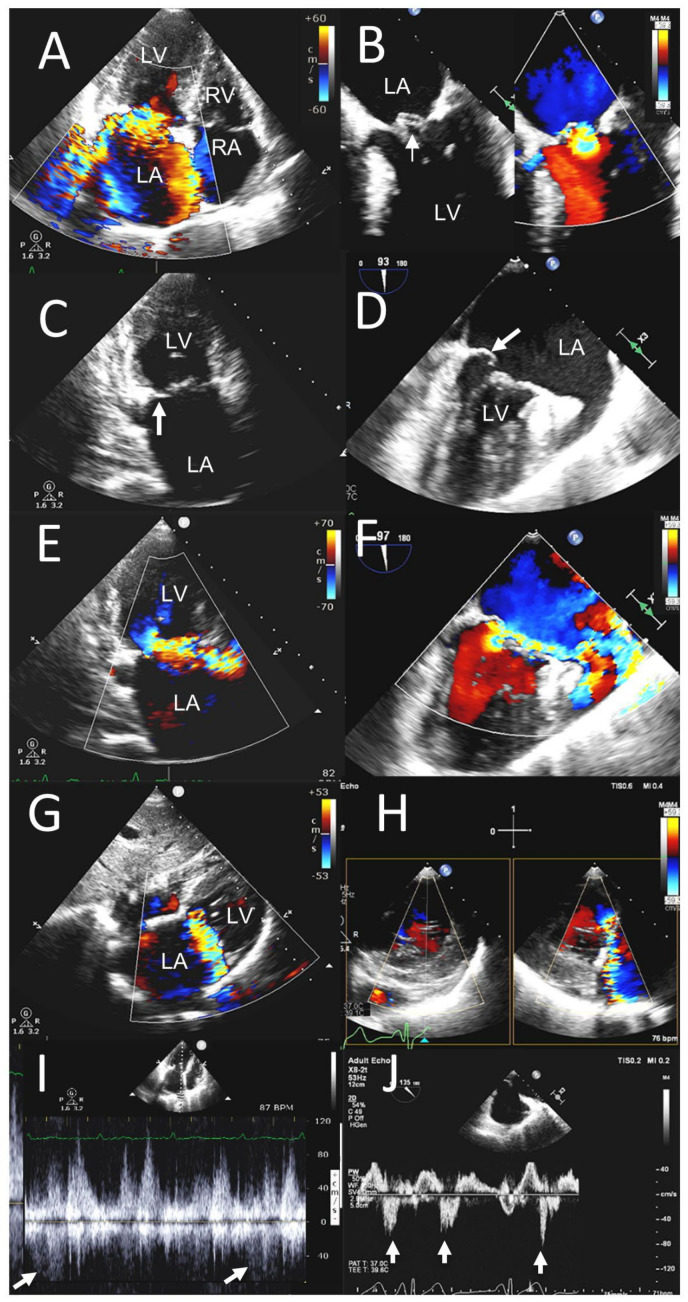
POCUS in assessment of MR severity and mechanism. An 86-year-old female was seen for a cardiac murmur and an episode of possible atrial arrhythmia. POCUS images (left-side panels) are shown compared to the TEE images (right-side panels). (**A**,**B**) Four-chamber views showing severe MR, with MR jet originating from medial commissural region and curling around the anterolateral LA wall (**A**), a flail A3 segment and proximal isovelocity surface acceleration (PISA) at a normal aliasing velocity of 59.3 cm/s (**B**). Severe LA enlargement is shown in both views. (**C**,**D**) Two-chamber views showing A3 prolapse (white arrow (**C**)) and flail A3 segment (white arrow (**D**)) along with dilated LA in (**C**,**D**). (**E**,**F**) Two-chamber color Doppler views showing an eccentric MR jet originating from medial commissural region and entering the LA appendage at aliasing velocities of 79 and 59.3 cm/s, respectively. (**G**) Subcostal four-chamber views showing MR jet originating from medial commissural region. (**H**) TEE biplane view showing PISA of MR jet from medial commissure at a normal aliasing velocity of 59.3 cm/s. (**I**) Apical four-chamber view with PW Doppler in the right upper pulmonary vein. (**J**) Mid esophageal view of the RA, interatrial septum and PW Doppler in the right upper pulmonary vein showing systolic flow reversal (white arrows (**I**,**J**)). LA, left atrium; LV, left ventricle; RA, right atrium; RV, right ventricle.

**Figure 4:**
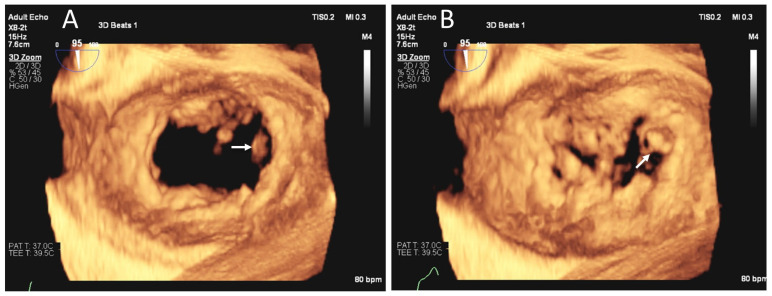
POCUS in assessment of MR severity and mechanism. Three-dimensional TEE image of the mitral valve from the atrial perspective in diastole (**A**) and systole (**B**) obtained using 3D zoom method in a single cardiac cycle showing flail medial commissural scallop (white arrows (**A**,**B**)).

On sTTE, there were 10 cases (13.0%) of ≥ moderate AR, 16 (20.8%) of ≥ moderate MR and 13 (16.9%) cases of ≥ moderate TR (Figure 1, Figure 2, Figure 3 and Figure 4 and Table 4). Among the 10 cases of ≥ moderate AR assessed on sTTE, 10 (100%) were detected on POCUS (Video S1). Among the other five patients presenting with mild-moderate AR detected on sTTE, one patient was considered to have moderate AR on POCUS. Among the 16 cases of ≥ moderate MR assessed on sTTE, 14 (87.5%) were detected correctly on POCUS (Video S2). Mitral valve prolapse and eccentrically directed jets of MR were detected on sTTE and POCUS in two patients. Two patients were post mitral intervention. Among the other nine patients presenting with mild-moderate (n = 8) or mild MR (n = 1) detected on sTTE, three patients were considered to have moderate MR on POCUS. Among the 13 cases of ≥ moderate TR assessed on sTTE, 12 (92.3%) were graded correctly on POCUS (Video S3). Among the other eight patients with mild-moderate TR detected on sTTE, two patients were considered to have moderate TR on POCUS. The sensitivity and specificity of POCUS were 90.9% and 100%, respectively, for detecting ≥ moderate AR, 82.4% and 96.7%, respectively, for ≥ moderate MR, and 85.7% and 98.4%, respectively, for ≥ moderate TR. The weighted κ coefficient between POCUS and sTTE was 0.94 (95% CI, 0.84–1.00) for the assessment of AR, 0.81 (95% CI, 0.65–0.97) for the assessment of MR, and 0.87 (95% CI, 0.72–1.0) for the assessment of TR.

### 3.3. ≥Moderate Valvular Stenosis

**Figure 5:**
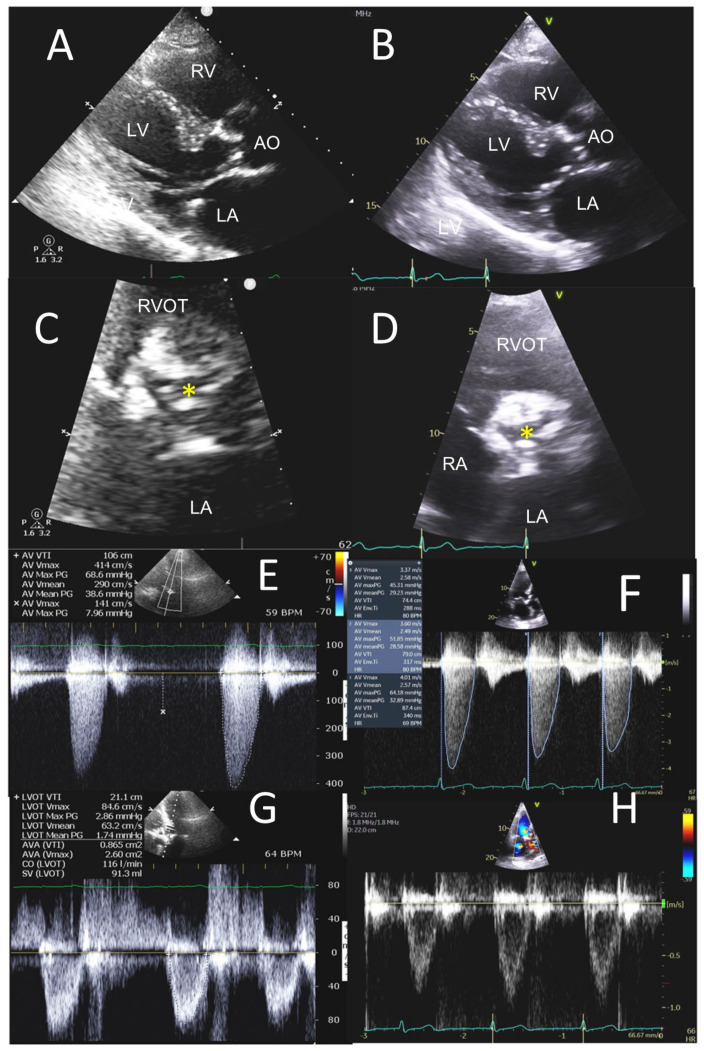
POCUS in stenotic aortic VHD. A 92-year-old male presented with exertional dyspnea and angina. POCUS images (left-side panels) are compared to the sTTE images (right-side panels). Parasternal long-axis views (**A**,**B**) show calcified aortic valve leaflets with restricted leaflet opening in systole parasternal short-axis views. (**C**,**D**) Heavily calcified aortic valve with marked restriction in leaflet opening and reduction in aortic valve orifice area (yellow asterisks (**C**,**D**)). CW Doppler in the apical five-chamber view (**E**) showed a mean gradient of 39 mmHg on POCUS, and mean gradient of 36 mmHg in the three-chamber view on sTTE (**F**). Aortic valve area was 0.87 cm^2^ on POCUS (**G**) and 0.88 cm^2^ on sTTE (**H**). Ao, aorta; LA, left atrium; LV, left ventricle; RA, right atrium; RV, right ventricle; RVOT, right ventricular outflow tract.

**Figure 6:**
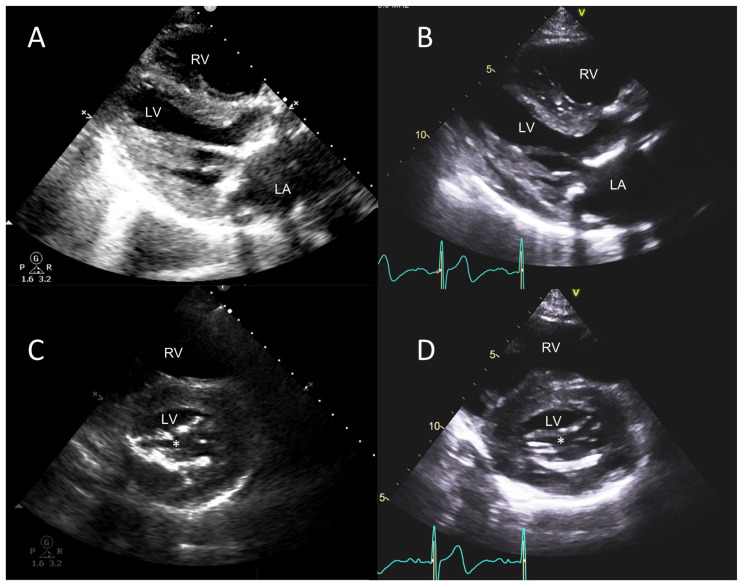
POCUS in stenotic mitral VHD. A 85-year-old male with history of biprosthetic aortic valve implant 8 years ago presented with dyspnea on exertion. POCUS images (left-side panels) are compared to the sTTE images (right-side panels). Parasternal long axis views (**A**,**B**) showing marked calcification of mitral leaflets extending from aortic sub annular region, and of the mitral annulus as well as subvalvular apparatus. Parasternal short axis views (**C**,**D**) in diastole showing marked calcification and restriction of leaflet mobility and reduction in mitral orifice area (white asterisks) suggestive of significant calcific MS. Severe mitral annular calcification is also shown. LA, left atrium; LV, left ventricle; RV, right ventricle.

**Figure 7:**
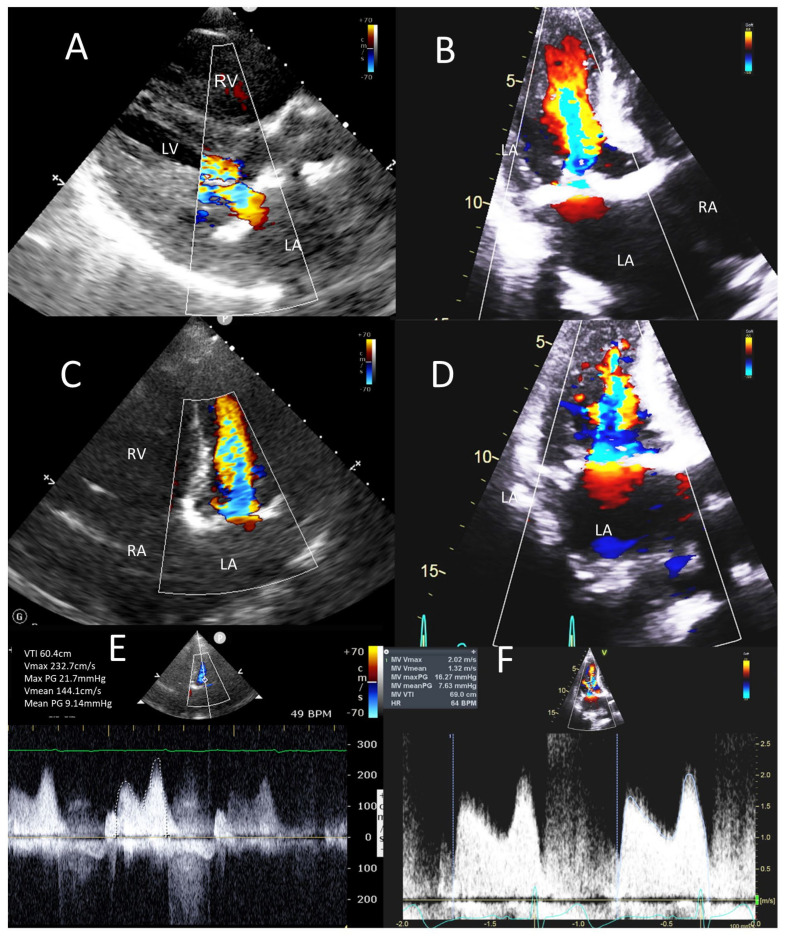
POCUS in stenotic mitral VHD. POCUS images (left-side panels) compared to the sTTE images (right-side panels) in the same patient as in Figure 6. Parasternal long axis view (**A**), apical 3 chamber view (**B**) and apical 4 chamber views (**C**,**D**) show turbulent mitral inflow on color Doppler. CW Doppler on mitral inflow in the 5 chamber view (**E**) and three chamber view (**F**) showed marked increase in mean mitral inflow gradient of 9 mmHg on POCUS (**E**) and 8 mmHg on sTTE (**F**). LA, left atrium; LV, left ventricle; RA, right atrium; RV, right ventricle.

On sTTE, 15 patients (19.5%) had ≥ moderate AS (Figure 5) and one patient (1.3%) had ≥ moderate MS (Figure 6 and Figure 7 and Table 4). Among the 15 cases of ≥ moderate AS assessed on sTTE, 15 (100%) were detected on POCUS (Video S4). Among the other three patients presenting with mild-moderate AS detected on sTTE, three patients were considered to have moderate AS on POCUS. Of the one case of ≥ moderate MS assessed on sTTE, one (100%) was detected on POCUS. Sensitivity and specificity in detecting ≥ moderate AS on POCUS were 83.3% and 100%, respectively. The weighted κ coefficient between POCUS and sTTE for the assessment of AS was 0.88 (95% CI, 0.76–1.0), indicating excellent agreement. Sensitivity and specificity were not calculated for MS, because there was only one available case. All of the severe AS (n = 5) patients subsequently received transcatheter or surgical aortic valve replacement (TAVR, n = 4; SAVR, n = 1).

### 3.4. Valve Repair and/or Valve Replacement

Thirty-four patients had valve repair and/or valve replacement identified on POCUS, and all were confirmed by sTTE: 13 cases (16.9%) with mitral valve replacement, 17 cases (22.1%) with TAVR/SAVR, three cases (3.9%) with tricuspid valve replacement/valvuloplasty and one case (1.3%) with pulmonary valve replacement. Five patients had significant residual paravalvular leak (PVL) (mitral PVL n = 2; aortic PVL n = 2) or aortic prosthetic stenosis (n = 1) on both POCUS and sTTE (Table 4) and received repeat surgery or intervention. Two of them had follow-up POCUS without residual PVL, subsequently confirmed on sTTE. The rest of the cases had normal function of prosthetic valve. Sensitivity and specificity in detecting prosthetic valve function on POCUS were 100% and 100%, respectively. The weighted κ coefficient between POCUS and sTTE was 1.0, indicating excellent agreement.

### 3.5. Other POCUS Findings

Sixty-one (79.2%) patients had PLAX-view images on POCUS. The measurement of LV chamber size and function on PLAX view (measured as part of this study) had good correlation with sTTE (LVEDD: 49 ± 10 mm vs. 51 ± 10 mm, r = 0.93, *p* < 0.0001; LVESD 36 ± 10 mm vs. 35 ± 10 mm, r = 0.92, *p* < 0.0001; LVEF: 55 ± 12% vs. 56 ± 11%, r = 0.95, *p* < 0.0001). Further, 45.7% (16/35) had dilated inferior vena cava and reduced collapsibility on POCUS. RVSP on POCUS (n = 27) had good correlation with sTTE (44 ± 11 mmHg vs. 38 ± 15 mmHg, r = 0.88, *p* < 0.0001). Additionally, 32.5% (n = 25) had assessment of PE. Of them, one had moderate PE confirmed by sTTE on reducing the dose of coumadin and discontinuing aspirin and had follow-up POCUS with trivial PE.

## 4. Discussion

In this study, we found an excellent agreement between POCUS and sTTE for the assessment of significant stenotic VHD. POCUS was highly sensitive and specific for accurate detection of significant regurgitant VHD of native valves. Furthermore, POCUS was able to evaluate normal or abnormal prosthetic valve function. POCUS was also able to detect the mechanism of native and prosthetic mitral and prosthetic aortic valve regurgitation. POCUS imaging performed by an echocardiologist required an average of 11 min. In our study, most patients presented with ≥ moderate MR or AS or history of TAVR/SAVR. Early detection of severe AS allowed surgical or percutaneous intervention or close follow-up to determine progression. POCUS can be incorporated during physician consultation with some extension of consultation time. Early referral for transesophageal echocardiography (TEE) and surgical and percutaneous valve repair/replacement expedited clinical management.

VHD is an increasingly common problem in routine clinical practice and a leading cause of mortality and morbidity. In industrialized countries, the main etiology of VHD is degenerative VHD [5,6], and mortality of VHD has increased over the past decades. In the United States, VHD accounted for 10% to 20% of all cardiac surgical procedures [13], and the mortality rate is increasing by 2.9% per year [14]. Considering that, screening for significant VHD using POCUS may help reduce mortality related to degenerative VHD.

POCUS has steadily grown over the past few decades and is increasingly applied in medical decision-making [8]. However appropriate training within the scope of practice of providers in various specialties is essential. Recognizing the value of proficiency in POCUS, the American College of Emergency Physicians recommends POCUS exams during emergency medicine residency [15]. To highlight the important role of POCUS in critical care, the National Board of Echocardiography has recently developed a certification pathway for critical care echocardiography [16]. Given its growth, international societies have published best practice recommendations and position statements to ensure adequate training, education, and quality control for the use of ultrasound in the clinical setting [17]. Similarly, the European Association of Echocardiography encourages this scanning to add information to the physical examination [18].

In our study, POCUS required less than a mean of 15 min (11 ± 9 min) to collect 44 images (±22 images) for every outpatient with quantitative assessment and measurement. Compared with sTTE, the assessment of significant VHD had high sensitivity and specificity and good agreement. The measurement of LV chamber size and function and RVSP on POCUS had good correlation with sTTE. Although screening using sTTE is the most reliable examination, it suffers from organizational and procedural delays as well as cost constraints. POCUS is free of these constraints and has excellent specificity and sensitivity in the detection of VHD in expert hands and performed according to a standardized but restricted scanning protocol to add information to the physical examination.

### 4.1. POCUS Studies in VHD

#### 4.1.1. POCUS with Handheld Units

Several studies have already shown the usefulness of POCUS with handheld units for the early detection of rheumatic VHD in endemic areas, with great sensitivity and specificity [19,20,21]. Recently, a study by Kikoȉne et al. demonstrated that POCUS performed by physicians with level III competency in echocardiography is reliable for identifying significant VHD and should be proposed as a new screening tool [9]. But the number of patients with ≥ moderate VHD in the study was small, and patients with right VHD and history of valvuloplasty and/or valve replacement were not included. Mild to moderate rhematic VHD may be under- or over-diagnosed utilizing handheld units in a population screening setting [19,21]. Over-diagnosis can reduce the specificity of POCUS [21]. POCUS with handheld units is far superior to physical examination by cardiologists [9,22] and may lead to a decrease in further diagnosis testing when the findings are normal [22]. Considering that POCUS significantly improves the bedside diagnosis after a relatively short training period and growing evidence of its feasibility, the trend of incorporating POCUS into the curriculum of medical schools and demand for integration of POCUS training in medical schools are on the rise [23].

#### 4.1.2. POCUS with Portable Small Ultrasound Machines

Several POCUS studies with portable small ultrasound machines demonstrated diagnostic accuracy and benefit in detecting significant cardiac pathology [23,24,25,26]. In our study, we demonstrated that POCUS was highly sensitive and specific for accurate detection of left VHD. Our study showed that POCUS also had high sensitivity and specificity for significant VHD from tricuspid regurgitation or for the evaluation of prosthetic valves. In our study, POCUS was performed by an expert echocardiologist, and our results, therefore, may be biased in favor of POCUS compared to cardiac POCUS performed by non-cardiologists or non-echo novices or experts. To improve the value of POCUS in clinical practice, it is of utmost importance to improve the level of competency of physicians, non-cardiologists as well as cardiologists and thus the accuracy of POCUS in the real-world setting.

Our study is unique in that it was performed in a real-world clinical setting. Earlier studies comparing POCUS with sTTE have been performed in a nonclinical setting, with POCUS and sTTE imaging performed among patients referred to the echo lab for sTTE [9] and with hypothetical treatment decisions [27].

Lower image resolution on a smaller ultrasound platform used in the study compared to sTTE may have caused overdiagnosis of valve regurgitation and stenosis in our study. The need to enter patient demographics, electrocardiogram leads, imperfect lighting and ergonomics, limited views and a limited imaging time contributed to sub-optimal image acquisition compared to sTTE. Visual grading for valve regurgitation may have falsely increased regurgitation severity, although significant VHD was measured by objective criteria.

Medication changes were performed at the clinical visit, which may have altered some findings [28]. In addition, there were time gaps between POCUS and sTTE, as well as clinical changes in patients during that time.

In our study, we found that significant VHD was over-diagnosed on POCUS, and this was possibly due to different definitions of grade severity for significant VHD (at least mild). We also observed one case where the time gap between POCUS and sTTE was almost 6 months, and the patient experienced disease progression, eventually receiving TAVR within the next 6 months.

Furthermore, other findings, such as LV chamber size and function, were detected in this study and had good correlation with data obtained by sTTE. Detection of cardiac chamber remodeling is crucial for the assessment and management of hemodynamically important valve lesions. Meanwhile, the presence and size of PE, as well as estimated right atrial pressure from inferior vena cava size and its respirophasic collapsibility, were also assessed in this study and helped to make medical and interventional treatment decisions.

## 5. Limitations

Visual estimation strongly relies on experience in echocardiography. Also, POCUS was examined by an expert physician, and image quality of POCUS was adequate in all 77 patients. However, the image quality of the POCUS may not be enough for less-experienced physicians/technicians, especially in patients with chronic obstructive pulmonary disease or obesity. The ultrasound machine used to perform POCUS is a cart-based portable US system capable of 2D, color Doppler and spectral Doppler. In addition the basic calculation package allowed for quantitative assessment of valve regurgitation severity including vena contracta, proximal isolvelocity flow acceleration, and velocities and gradients of regurgitant and stenotic lesions using continuity equations. Handheld POCUS devices do allow linear measurements. Color Doppler, which is integrated into POCUS devices, generally allows for differentiation of mild from severe regurgitant lesions accurately, even in the absence of spectral Doppler [27]. There is no continuous Doppler in current handheld POCUS devices, which prevents the quantification of flow velocities and hence limits the ability to grade the severity of regurgitant and especially stenotic lesions. More recently, PW Doppler can now be performed in most handheld devices. At present, the Kosmos device (Kosmos, U2i), which is in between handheld units and small cart-based ultrasound units like the one used in our study in size, has 2D, M-mode, color Doppler, CW and PW Doppler capabilities, as well as the ability to quantitate significant VHD [26].

## 6. Conclusions

POCUS can identify patients with significant VHD and may serve as a powerful screening tool for VHD to expedite clinical decision-making in clinical practice.

## Figures and Tables

**Table 1:** Clinical characteristics of the study population (all vital sign data are from POCUS visit).

	Overall Cohort (*n* = 77)
Age, years	72 ± 11
Male, n (%)	45 (58.4)
BMI, kg/m^2^	26.5 ± 4.9
Heart rate, beats/min	69 ± 15
Systolic blood pressure, mmHg	135 ± 22
Diastolic blood pressure, mmHg	73 ± 11
Hypertension	61 (79.2)
HF	37 (48.1)
DM	10 (13.0)
COPD	8 (10.4)
AF	25 (32.5)
CAD	37 (48.1)
Stroke/TIA	9 (11.7)
Renal insufficiency	27(35.1)
Cancer	9 (11.7)
GFR, ml/min/kg	60 ± 18
Hemoglobin, g/dl	10.3 ± 3.2
Creatinine, mg/dL	1.4 ± 1.8
NT-proBNP, pg/Ml ^a^	828 (182,1868)

Data as mean ± standard deviation, numbers (percentages) or median (interquartile range); Abbreviations: AF, atrial fibrillation; BMI, body mass index; CAD, coronary artery disease; COPD, chronic obstructive pulmonary disease; DM, diabetes mellitus; GFR, glomerular filtration rate; HF, heart failure; NT-proBNP, N-terminal pro b-type natriuretic peptide; TIA, transient ischemic attack. ^a^ n = 27 (35.1%).

**Table 2:** sTTE echocardiographic parameters.

	Value
Body surface area, m^2^	1.9 ± 0.3
IVS, mm	11 ± 2
PW, mm	10 ± 2
LV end-diastolic diameter, mm	51 ± 10
LV end-systolic diameter, mm	35 ± 10
LVEF, %	58 ± 11
LVEDV, ml	125 ± 55
LVESV, ml	56 ± 38
LAVi, mL/m^2^	46 ± 20
TR velocity, m/s	2.7 ± 0.5
RVSP, mmHg	38 ± 15

Values are mean ± standard deviation. Abbreviations: IVS, interventricular septum; LAVi, left atrium volume index; LV, left ventricle; LVEDV, left ventricular end-diastolic volume; LVESV, left ventricular end-systolic volume; PW, posterior wall; TR, tricuspid regurgitation; RVSP, right ventricular systolic pressure.

**Table 3:** Treatment initiated or referral after POCUS.

	Number of Patients (%)
Standard TTE/TEE/Stress echocardiography	24 (31.2)
Other tests (cardiopulmonary exercise test, contrast CT)	5 (6.5)
Initiate/increase/decrease/discontinue diuretics	25 (32.5)
Other medications (antihypertension, antiarrhythmic drugs, beta-blocker, anticoagulation, etc.)	10 (13.0)
TEE guide cardioversion	1 (1.3)
Cardiac rehabilitation	2 (2.6)
Right/left heart catheterization	6 (7.8)
Surgical or percutaneous intervention	17 (22.1)
Follow-up (no progression, normal function of prosthetic valve)	15 (19.5)

Values are n (%). Abbreviations: TTE, transthoracic echocardiogram; TEE, transesophageal echocardiogram; CT, computerized tomography.

**Table 4:** Significant VHD (≥moderate) diagnosed on sTTE and POCUS.

	sTTE	POCUS
≥moderate valvular heart disease, n (%).		
Mitral regurgitation	16 (20.8)	17 (22.1)
Mitral stenosis	1 (1.3)	1 (1.3)
Aortic regurgitation	10 (13.0)	11 (14.3)
Aortic stenosis	15 (19.5)	18 (23.4)
Tricuspid regurgitation	13 (16.9)	14 (18.2)
Valve function abnormalities, n (%)		
Paravalvular leak on MV	2 (2.6)	2 (2.6)
Paravalvular leak on AV	2 (2.6)	2 (2.6)
Aortic prosthetic valve stenosis	1 (1.3)	1 (1.3)

Values are n or n (%). Abbreviations: MV, mitral valve; AV, aortic valve.

## Data Availability

The data presented in this study will be available upon request based on institutional policy.

## Supplementary Materials

The following supporting information can be downloaded at: https://www.mdpi.com/article/10.3390/jcm12206474/s1, Video S1: POCUS in aortic regurgitation. Zoomed parasternal long-axis view obtained during POCUS. Central mild to moderate appearing AR jet is shown. Video S2: POCUS in moderate tricuspid regurgitation. Apical four-chamber view obtained during POCUS. Central jet of moderate TR is shown along with atrial enlargement and right ventricular enlargement. Video S3: POCUS in assessment severity and mechanism of mitral regurgitation. Apical four-chamber view obtained during POCUS in a patient with dyspnea on exertion post mitral valve repair with edge-to-edge repair using two mitral clips. Severe eccentric posteriorly directed MR jet is shown curling around the dilated left atrium and causing a pulsatile left atrium in systole. Video S4: POCUS in aortic stenosis. Zoomed aortic valve color Doppler view in the transverse plane from parasternal short-axis window during POCUS showing systolic flow across the narrow aortic valve systolic orifice.

## Abbreviations

AR	aortic regurgitation
AS	aortic stenosis
AVR	aortic valve replacement
MR	mitral regurgitation
MS	mitral stenosis
POCUS	point-of-care ultrasound
sTTE	standard transthoracic echocardiography
TR	tricuspid regurgitation
VHD	valvular heart disease
